# Mining Methylation for Early Detection of Common Cancers

**DOI:** 10.1371/journal.pmed.0030479

**Published:** 2006-12-26

**Authors:** Romulo M Brena, Christoph Plass, Joseph F Costello

## Abstract

A single method that detects multiple common cancer types at an early stage would have the biggest payoff for cancer control, say Brena and colleagues.

Cancer is one of the most common causes of death worldwide. Lung cancer is the leading cause of cancer-related death worldwide [1], with a five-year survival rate of 10% in Europe and 15% in the United States [2,3]. Strikingly, for lung cancers detected in their early stage, the rate of recurrence within five years is less than 50%. When detected early, current therapies often cure common cancers, including those of the lung, breast, colon, rectum, stomach, and prostate [4]. Therefore, the development of reliable, noninvasive, and cost-effective early detection methods for common cancers is a priority of translational cancer research. Theoretically, a single method that detects multiple common cancer types at an early stage would have the biggest payoff for cancer control.

## The Ups and Downs of DNA Methylation in Cancer

Biomarkers of cancer cells have been derived from the genetic mutations and epigenetic alterations, such as DNA methylation, that together transform normal cells into tumors. DNA methylation involves the addition of a methyl group to sites where cytosine is followed by guanine along the DNA (i.e., a CpG dinucleotide, with p indicating the phosphate backbone that connects the cytosine and guanine nucleotides), and is essential for chromosome stability, maintenance of gene expression states, and proper telomere length in normal cells [5]. In primary human tumors, however, DNA methylation patterns are severely disrupted. This disruption includes aberrant gain of DNA methylation at CpG islands (short stretches of DNA with an abundance of CpG dinucleotides, located in gene regulatory regions) and loss of methylation in single copy and repetitive sequences. While CpG island methylation is associated with gene silencing, hypomethylation can result in gene activation and chromosomal instability [6].

There remains a potentially vast, untapped resource for cancer-specific methylation biomarkers.

The subset of CpG islands subjected to aberrant methylation in primary human tumors is non-random, due in part to selection of gene silencing events that favor unregulated cell growth, and also due to intrinsic sequence properties that may underlie gene-specific susceptibility to methylation [7–10]. One hypothesis is that alterations in DNA methylation play a key role in tumor initiation, and if this is true, methylation markers are ideally suited for detecting cancer in the early stages [11,12].

In contrast to tumor type–specific methylation markers, pan-cancer methylation markers could detect a far greater number of early stage cancers. Initial results of a prospective study of such markers are encouraging [13]. Belinsky and colleagues used aberrant methylation in a six-gene panel to detect lung cancer in sputum samples taken months to years prior to the clinical onset of cancer [13]. Other bodily fluids are similarly useful for detection of noninvasive cancer via methylation markers, such as urine for kidney, bladder, or prostate cancer, and serum or nipple aspirates for breast cancer [14,15]. Notably, the panels of aberrantly methylated genes in these different tumor types overlap significantly, indicating that assays for pan-cancer methylation markers are already available for testing. The important role of aberrant methylation in cancer detection and prognostication has been established on a small proportion of the CpG island–containing genes. Thus, there remains a potentially vast, untapped resource for cancer-specific methylation biomarkers.

## A New Study of Pan-Cancer Methylation Markers

In a new study published in *PLoS Medicine*, Shames et al. aim for a big payoff by searching genome-wide for CpG island methylation markers characteristic of not just one type of cancer, but common to several cancers of epithelial origin [16]. Identification of such markers could facilitate detection and diagnosis, and might also shed light on molecular pathways that are characteristic of tumorigenesis in general, potentially providing new clinically relevant therapeutic targets with more widespread application.

Shames and colleagues began the quest for a pan-cancer marker by gene expression profiling of non-small cell lung cancer cell lines before and after treatment with the DNA methylation inhibitor 5-aza-2′-deoxycytidine. Their initial goal was to find genes expressed in a normal cell from which the tumor might arise, namely human bronchial epithelial cells, but silenced in the cancer cell lines and reactivated by inhibition of DNA methylation. The initial experiment netted 132 tumor-specific candidates. Winnowing this gene subset to a manageable 45 genes involved random selection, and was followed by validation of gene silencing in primary lung tumors relative to the tumor-free adjacent lung.

The eight most frequently methylated genes obtained from the lung cancer data were then tested in a set of 109 tumors from breast, colon, and prostate. Four genes, BCN1, MSX1, CCNA1, and ALDH1A3, showed extensive DNA methylation in all four epithelial cancers. In particular, BNC1 and MSX1 were highly sensitive and specific for tumor detection.

The authors concluded that key pathways altered epigenetically in the tumorigenic process may be shared across cancers of epithelial cell origin, despite obvious differences in their tissue source. This finding is of importance, since it highlights the possibility of identifying a common epigenetic denominator acting across tumor types, and perhaps underlying malignant transformation in general.

However, we must again consider that these genes may be susceptible to aberrant DNA methylation, which could be due in part to their primary DNA sequence. Thus, it remains to be determined if the high frequency of DNA methylation observed in the BCN1, MSX1, CCNA1, and ALDH1A3 genes stems from a functional need to abrogate expression and thus contribute to tumor initiation and/or progression, from an intrinsic susceptibility of these loci to aberrant DNA methylation, or both. In either case, their utility as markers of cancer cells will be unaffected.

## Cautions and Clinical Implications

One hazard of using DNA methylation as a marker of cancer is the distinct possibility of false positives. The perfect marker would detect all cancer cases (100% sensitivity) and would not mistake normal cells for cancer cells (100% specificity). However, the influence of ageing, diet, or hormones on DNA methylation may confound results if even a minor fraction of normal cells are methylated at the gene of interest in cancer-free individuals. This cautionary note has been sounded by Shames and colleagues [16] and by others [17]. The problem is that an assay sufficiently sensitive to detect a rare cancer cell in blood or bodily fluids could be particularly susceptible to this pitfall. Nevertheless, developing a single routine test for major cancer types in at-risk or asymptomatic individuals, paired with follow-up tests for specific malignancies, is a goal of high priority.

Efforts toward the perfect universal DNA methylation marker for early detection of tumors are well underway in research laboratories worldwide. Epigenetics researchers from Asia, Europe, and the US are joining forces to map the entire epigenome of normal and cancer cells [18]. Assays are currently available to detect aberrant DNA methylation in samples such as sputum, blood, feces, urine, and nipple aspirates, which can be procured via minimally invasive procedures and are likely to contain tumor cells and tumor DNA shed from a primary tumor mass [14,15].

Research endeavors such as these of Shames et al. are pivotal for the first phase of identification of suitable markers. Technological advances, such as the development of DNA methylation arrays, will undoubtedly aid in the methylation marker discovery phase. The next phase will include testing markers retrospectively and then prospectively in clinical settings, in a high-throughput and cost-effective manner. Coupling the discovery of new DNA methylation markers with investigation into their functional relevance will benefit early detection efforts and improve our understanding of the tumorigenic process.

Currently recommended methods for early detection of cancer include spiral computed tomography for patients at risk for lung cancer, breast exams and mammography for breast cancer detection, fecal occult blood tests and colonoscopy for colon cancer, and endoscopy for gastric cancers. However, several of these methods are expensive, prohibiting large-scale population-based use. It remains to be seen whether the new suite of DNA methylation markers from Shames et al. or those from prior studies will be true pan-cancer markers, and most importantly, whether they will outperform existing methods for early detection.
